# Successful TB treatment outcome and its associated factors among TB/HIV co-infected patients attending Gondar University Referral Hospital, Northwest Ethiopia: an institution based cross-sectional study

**DOI:** 10.1186/s12879-017-2238-7

**Published:** 2017-02-08

**Authors:** Yenework Sinshaw, Shitaye Alemu, Abel Fekadu, Mucheye Gizachew

**Affiliations:** 1 University of Gondar referral Hospital, Gondar, Ethiopia; 2 Department of Epidemiology and Biostatistics, University of Gondar, Institute of Public Health, Gondar, Ethiopia; 3 University of Gondar, College of Medicine and Health Science, School of Biomedical Sciences, Gondar, Ethiopia

**Keywords:** Successful treatment outcome, TB/HIV co-infected patients, Tuberculosis, Ethiopia

## Abstract

**Background:**

Tuberculosis/Human immunodeficiency virus (TB/HIV) co-infection is bidirectional and synergistic which mainly affects interventions that have been taken on the area. Tb patients co-infected with HIV have poorer treatment outcome as compared to non-co-infected patients. There is limited information regarding successful TB treatment outcomes and its associated factors; a reason that this study was planned to investigate.

**Methods:**

An institution based cross sectional study was carried out from July 2010 to January 2016. Data were abstracted from patients’ medical chart using data abstraction format. The completeness of the data was checked and cleaned manually. Then, it was entered and analyzed by using SPSS version 20.0. Bi-variable and Multi-variable logistic regression model was fitted to identify factors associated with successful Tb treatment outcome. Significance was obtained through adjusted odds ratio with its 95% CI and a *p* < 0.05.

**Results:**

Successful TB treatment outcome among TB/HIV co-infected patients in Gondar University Hospital was 77.3% [95%CI 72.6–81.9]. Being residing in outside the Gondar town [AOR = 0.44, 95%CI: 0.25–0.80], having less than the mean baseline weight (<43.7 kg) at initiation of TB treatment [AOR = 0.51, 95% CI: 0.29–0.89], being in the bedridden condition [AOR = 0.23, 95% CI: 0.1–0.23], and experiencing anti-TB treatment side effect [AOR = 0.35, 95% CI: 0.12–0.98] were the factors that resulted the patient in treatment failure.

**Conclusion:**

Successful Tb treatment outcome among TB/HIV co-infected patients was lower than the target set by Global Plan to Stop TB 2011–2015. Strengthening collaborative TB/HIV management activities that would trace the identified factors shall be recommended to increase successful treatment outcome of TB.

## Background

Despite various efforts made to prevent and/or control the disease, many countries have not yet achieved the TB control targets. Rather, it remains a major public health problem leading to mortality in high HIV- burden countries [1, 2]. HIV drives TB epidemic in many countries, especially in the sub-Saharan Africa, where 82% of the world’s TB/HIV co-infection exists [3]. TB/HIV co-infection in one’s body potentiate one another and hastening the weakening of the host’s immunological functions. HIV co-infection is the most important risk factor for developing active TB, which increases susceptibility to primary infection, re-infection and/or reactivation for patients with latent TB. TB also has a negative impact on the immune response to HIV, increasing the progression from HIV infection to acquired immunodeficiency syndrome (AIDS) [4].

Tuberculosis/HIV co-infection constitutes several problems including diagnostic and therapeutic challenges in the healthcare settings [5–7]. It is supported by a study conducted in USA that revealed treatment of TB in co-infected patients differ from those patients who are infected with TB only [8]. Some of these challenges: clinical problems about duration of treatment, frequency of drug administration, pill burden, management of drug interactions, and complications of therapy like drug toxicity and immune reconstitution inflammatory syndrome (IRIS). Since such patients are being treated for two infectious diseases, the goals of treatment for both must be balanced through therapy integration, use of concurrent Antiretroviral Therapy (ART), prevention of HIV-related co-morbidities, controlling drug toxicity, and monitoring of IRIS [2, 4]. This would bring optimal outcomes in terms of treatment response and prevention of drug resistance.

Among the 22 high TB burden countries, Ethiopia ranks 7^th^ with an incidence rate of 379 cases of all forms of TB per 100,000 populations. Continuous tracing of TB treatment outcome, mainly in those TB/HIV co-infected patients, is essential in the area [9, 10]. However, in our study site, there was scarcity of recent data describing successful TB treatment outcome and its associated factors in TB/HIV co-infected patients [11]. Understanding its treatment outcome and associated factors in such a population may help in better management of TB. Therefore, this study assessed the successful TB treatment outcomes and its associated factors among TB/HIV co-infected patients attending the TB DOTS Clinic at the Gondar University Hospital, Northwest Ethiopia.

## Methods

### Study setting and design

The study was conducted at the Gondar University Hospital, Northwest Ethiopia, situated 738 kilometers far away from the capital. The Hospital serves over five millions of people from its catchment area (Amhara National Regional State Bureau of Health, Health Research Thematic Areas of Amhara Regional Health Bureau: Amhara Regional Health Bureau Health Research and Technology Transfer Core Process in collaboration with Ethiopian Network for HIV/AIDS Treatment Care and Support (ENHAT-CS) Amhara Region Program, Unpublished). An instituttion based cross-sectional analysis of successful TB treatment outcome among TB/HIV co-infected patients registered from July 2010 to January 2016 was conducted.

Gondar University hospital uses different types of TB diagnostic techniques like AFB microscopy or smear, X-ray, pathological, and Xpert MTB/RIF as required procedurally. TB DOTS clinic serves both adult and pediatric age groups with free drugs like Rifampicin, Isoniazid, Pyrazinamide and Ethambutol (2RHZE) for 2 months intensive phase with daily observation, and Rifampicin and Isoniazid (4RH) for 4 months continuous phase with monthly follow up for new TB patient. The hospital also provides Rifampicin, Isoniazide, Pyrazinamide, Ethambutol and Streptomycin (3RHZES) for 3 months intensive phase/, and Rifampicin, Isoniazide and Ethambutol (5RHE) for 5 months continuous phase for retreatment patients. For TB/HIV co-infected patients; they should take daily for 6 months; 2 months intencive phase taking Rifampicin, Isoniazid, Pyrazinamide and Ethambutol (2RHZE) and 4 months continuation phase taking Rifampicin and Isoniazid (4RH).

All Tb patients under treatment follow up were tested for HIV. For HIV screening, nationally approved rapid serological testing algorisms (KHB → StatPak → Uni-gold) were used. As soon as HIV is identified in a TB patient, the patient is enrolled to ART clinic and co-trimoxazole preventive therapy (CPT) started. The national TB/HIV implementation guideline recommends provision of ART for TB/HIV co-infected individuals if they are WHO clinical stage IV or CD4 < 350/mm.

### Study population and sampling procedure

The study population was all TB/HIV co-infected patients who attended at the Gondar University Hospital. TB/HIV co-infected patients who were transferred out to other health institution during the study period were excluded. The necessary sample size (n) was computed by single population proportion formula ([*n* = [(Z_a/2_)^2^*P (1-P)]/d^2^]) assuming 95% confidence level of Z a/_2_ = 1.96, 5% margin of error, and proportion (p) of 70.9% according to the previous similar study from Tigray region [12]. The calculated sample was 318 and by using correction formula the final sample size was reduced to 203. However, all TB/HIV co-infected patients’ record at the Gondar University Hospital who started DOTs and had complete medical records on TB treatment outcome in the past 5 years were included in the study.

### Data collection and variable measurement

Data were collected using pre-tested and updated data abstraction format. The format was developed by considering variables to be studied that found in the patient’s TB/HIV treatment follow up chart and/or hospital registration book. Data collectors had a brief training that emphasized on inclusion and exclusion criteria of the study participants, and recording of the right information from the hospital registration book and/or patient’s follow-up medical chart.

Clinical case and treatment outcome definitions were used according to the standard definitions of National Tuberculosis and Leprosy Control Program (NTLCP) [12] and WHO guideline [13]. Treatment outcomes were divided into (cured, completed treatment, defaulted/interrupted, failed, died, and not evaluated). Successful/favorable/good outcome were considered for TB patients who were cured (i.e., negative smear microscopy at the end of treatment and on at least one previous follow-up test) and/or completed treatment or sum of cases that cured and completed treatment [14].

Unsuccessful/unfavorable/poor outcome was considered for TB patients resulted in treatment failure, default or death [14]. Patients with documented treatment completion and resolution of symptoms, but not sputum smear microscopy available at the end of treatment were considered as completed treatment. Defaulted/Interrupted patient was a patient whose treatment was interrupted for two consecutive months or more for any reason without medical approval.

Patients with clinical and/or bacteriological signs of continued active disease or deterioration requiring a treatment change were considered as failed patients. Died patient is a patient who died for any reason during the course of treatment. A patient whose treatment outcome is unknown (including former “transfer out”) was considered as not evaluated patients. A Patient who smoked at time of TB diagnosis or had history of cigarette smoking were taken as smokers and a patient who had never smoked in his/her life [15, 16] were taken as non-smokers.

Functional status was determined based on WHO guideline as follows; (a) Working = able to perform usual work in or out of the house, harvest, go to school or, for children, normal activities or playing (b) Ambulatory = able to perform activities of daily living but not able to work or play, and (c) Bedridden = not able to perform activities of daily living. Tb treatment side effect was ascertained by assessing and/or following of the patients’ complain, and a comprehensive clinical history and additional medical exams along with different laboratory investigations including chemistry and hematocrit analysis.

Socio-demography of the patient (age, sex, economic status), concomitant condition (World Health Organization clinical staging, functional status), Biological factor (CD4+ Count), Patient Behavior (Smoking history), and medication related factor (History of TB treatment, drug side effect, ART) were collected as an independent variables of the study.

### Data quality

Data abstraction checklist was pre-tested at Felege Hiwot Referral Hospital, which provides similar services to the TB/HIV co-infected patients prior to the commencement of the actual data collection. Data were collected by the trained data collectors with close supervision and assistant of the investigators. Completeness of the data was checked, coded and entered into the computer using SPSS version 20 statistical software on a daily basis. Each entry was cross checked independently to ensure the quality of data.

### Data analysis

A total of 324 TB/HIV co-infected patients were primarily enrolled in the study. Of this figure, 308 patients fulfilled the inclusion criteria and were analyzed. Descriptive statistics (mean (SD), proportion) aimed to summarize patients’ characteristics across the outcome variables was used. Findings were described using words and tables. Association between successful TB treatment outcomes and each independent variable was analyzed using Bi-variable and Multi-variable logistic regression model.

The outcome was categorized as successful (cure + treatment completed) and unsuccessful (default, failure, death) treatment outcomes [14, 17]. All variables were entered into multivariable logistic regression using backward LR method to control confounding effect. Explanatory variables which had significant association with the outcome at *p*-value less than 0.2 in the bi-variable binary logistic regression were entered to multivariable logistic regression model to identify the predictors of successful Tb treatment outcome. Association between outcome and predictor variables was calculated using Adjusted odds ratio at *p*-value <0.05 and 95% confidence interval. Assumption of goodness of the model was checked by Hosmer-lemeshow test (*p* = 0.828).

### Ethical consideration

Ethical approval was obtained from the Ethical Clearance Committee of the School of Medicine, College of Medicine and Health Sciences, University of Gondar prior to the commencement of the study. Confidentiality of the participants’ information was maintained by giving participant’s code number and the data locked in the cupboard and computer with password.

## Results

### Socio-demographic and behavioral characteristics of study participants

A total of 308 TB/HIV co-infected patients record were analyzed in this study. Of these, 175 (56.8%) were male. The mean age of the respondents was 31.7 (SD ± 13.4) year. Two hundred and twenty nine (74.4%) study participants were residing in the Gondar town, and 206(66.9%) were ever married (married, divorced, or widowed) during their visits of the health institution. One hundred thirty three (43.2%) had primary educational level (Grade 1 to 8^th^), 97(31.5%) were daily laborer, and 264(85.7%) had no history of cigarette smoking (Table 1).

**Table 1:** Socio-demographic and behavioral characteristics of respondents, Gondar University Hospital, 2016

Variables	Categories		Frequency of TB treatment outcome, n (%)	
		Total	Successful, n (%)	Unsuccessful
Sex	Male	175	136 (77.7)	39 (22.3)
	Female	133	102(76.7)	31(23.3)
Age (years)	<15	42	29(69.1)	13(30.9)
	15–29	81	70(77.8)	20(22.2)
	30–44	137	107(78.1)	30(21.9)
	>44	48	30(81.1)	7(18.9)
Residence	Gondar town	229	186(81.2)	43(18.8)
	Out of Gondar town	79	52(65.8)	27(34.2)
Marital status	Ever married	206	164(79.6)	42(20.4)
	Unmarried	57	42(73.7)	15(26.3)
	Not applicable	45	32(71.1)	13(28.9)
Education	No education	60	49(81.7)	11(18.3)
	Primary	133	97(72.9)	36(27.1)
	Secondary and above	100	82(82.0)	18(18.0)
	Not specified/NA)	15	10(66.7)	5(33.3)
Occupation	Farmer	27	20(74.1)	7(25.9)
	Merchant	46	31(67..4)	15(32.6)
	Housewife	43	37(86.0)	6(14.0)
	Student	37	27(73.0)	10(27.0)
	Employed	43	34(79.1)	9(20.9)
	Daily laborer	97	80(82.5)	17(17.5)
	Note specified	15	9(60.0)	6(40.0)
Cigarette smoking	Non-smoking history	264	206(78.0)	58(22.0)
	Smoking history	19	15(58.9)	4(21.1)
	Not specified	25	17(68.0)	8(32.0)

### Concomitant, biological and medication related factors of the study participants

One hundred and ninety five (63.3%) respondents had <200 CD4 cell/mm^3^ at initiation of their TB treatment. The mean baseline weight at entry of TB treatment was 43.7 kg. Six hundred sixty three (52.9%) of the study participants have been found to be on working functional status. Two hundred seventy four patients (88.9%) were on Co-trimoxazole Prophylactic Treatment (CPT), 188(61.0%) on ART, and 188(61.0%) were categorized as WHO clinical stage III during the initiation of their TB treatment. In addition, 231(75.0%) respondents had not history of TB treatment, 219(71.1%) had Pulmonary tuberculosis (PTB), and 290(94.2%) had no TB treatment side effects (Table 2).

**Table 2:** Concomitant, biological and medication related factors of the study participants, University of Gondar, 2016

		Total	Tb treatment outcome, *n* (%)	
Variables/factors	Categories	*N* = 308	Successful	Unsuccessful
CD4+ count at TB treatment entry	<200 cells/mm^2^	195	149(76.4)	46(23.6)
	≥200 cells/mm^2^	113	89(78.8)	24(21.2)
Mean base line weight (kg) at TB treatment entry	<43.7	132	92(69.7)	40(30.3)
	≥43.7	176	146(83.0)	30(17.0)
Functional status	Working	163	136(83.4)	27(16.7)
	Ambulatory	88	74(84.1)	14(15.9)
	Bedridden	57	28(49.1)	29(50.9)
INH prophylaxis	Yes	12	10(83.3)	2(16.7)
	no	296	228(77.0)	68(23.0)
CPT	Yes	274	214(78.1)	60(21.9)
	no	34	24(70.6)	10(29.4)
ART at TB treatment entry	pre-ART	188	143(76.1)	45(23.9)
	on ART	120	95(79.2)	25(20.8)
WHO staging	III	188	154(81.9)	34(18.1)
	IV	120	84(70.0)	36(30.0)
TB treatment history	Yes	77	59(76.6)	18(23.4)
	No	231	179(77.5)	52(22.5)
TB type	Pulmonary	219	175(79.9)	44(20.1)
	Extra-pulmonary TB	89	63(78.8)	26(29.2)
Category(case definition) of TB	New TB cases	221	169(76.5)	52(23.5)
	Relapse cases	48	37(77.1)	11(22.9)
	Transfer in	39	32(82.1)	7(17.9)
Smear status (*n* = 219)	Positive	47	38(80.9)	9(19.1)
	Negative	172	133(77.3)	39(22.7)
Treatment side effect	Yes	18	10(55.6)	8(44.4)
	No	290	228(78.6)	62(21.4)

### Successful TB treatment outcome of the study participants

As shown in Table 3, of the 308 TB/HIV co-infected patients who were included in this study, 238 (77.3%) (95% CI 72.6–81.9) had successful TB treatment outcome, in which, 32(10.4%) and 206(66.9%) have been cured and completed their TB treatment respectively (Table 3).

**Table 3:** TB treatment outcome of the study participants, University of Gondar, 2016

TB treatment outcome	Categories	Frequencies (n)	Percent (%)
Successful outcome	-	238	77.3
	Cured	32	10.4
	Completed	206	66.9
Unsuccessful outcome	-	70	22.7
	Defaulted	37	12.0
	Failed	2	0.6
	Death	31	10.1

### Factors associated with successful TB treatment outcome of the study participants

In the Multi-variable logistic regression analysis; residence, mean baseline weight (kg), functional status, and TB treatment side effect of the respondents were significantly associated with the successful TB treatment outcome (*P* < 0.05).

Accordingly, the odds of having successful Tb treatment outcome was 66% lower among TB/HIV co-infected patients who have been residing outside the Gondar town as compared to those who have been living in the Gondar town [AOR = 0.44, 95% CI: 0.25–0.80]. The Odds of having successful TB treatment outcome among TB/HIV co-infected patients having the mean base line weight <43.7 kg was 49% lower as compared to patients who had ≥ 43.7 kg at the initiation of their TB treatment [AOR = 0.51, 95% CI: 0.29–0.89]. Similarly, the Odds of having successful TB treatment outcome was 77% lower among patients on bed ridden as compared to patients on working condition[AOR = 0.23, 95% CI: 0.1–0.23]. Lastly, the odds of successful TB treatment outcome was 65% times lower among Tb/HIV co-infected patients who complained TB treatment side effects than their counterparts [AOR = 0.35, 95% CI: 0.12–0.98] (Table 4).

**Table 4:** Bi-variable and multi-variable logistic regression analysis of factors associated with TB treatment outcome of the study participants, University of Gondar, 2016

Variable	Categories	TB Treatment outcome, *n* = 308		COR (95% CI)	AOR (95% CI)
		Successful	Unsuccessful		
Sex	Male	136	39	1	
	Female	102	31	0.94 (0.55, 1.61)	
Age	<15	29	13	0.45 (0.16, 1.22)	
	15–29	59	22	0.54 (0.22, 1.32)	
	30–44	110	27	0.82 (0.34, 1.94)	
	>44	40	8	1	
Residence	Urban	186	43	1	1
	Rural	52	27	0.45 (0.25, 0.79)	0.44 (0.25, 0.80) *
Educational status	No education	49	11	1	
	Primary	97	36	0.61 (0.28, 1.29)	
	Secondary and above	82	18	0.10 (0.45, 2.34)	
	Not specified/NA)	10	5	0.45 (0.13, 1.58)	
Marital status	Ever married	164	42	1	
	Unmarried	42	15	0.72 (0.36, 1.42)	
	Not specified	32	13	0.63 (0.30, 1.31)	
CD4 at TB Rx	<200 cells/mm^3^	149	46	1	
	≥200 cells/mm^3^	89	24	1.15 (0.66, 2.00)	
Mean baseline weight (Kg)	<43.7	92	40	0.47 (0.28–0.81)	0.51(0.29–0.89) *
	>/=43.7	146	30	1	1
Functional status	Working	136	27	1	1
	Ambulatory	74	14	1.05 (0.52, 2.12)	1.29(0.62, 2.67)
	Bedridden	28	29	0.19 (0.09, 0.37)	0.24 (0.12, 0.48)*
CPT	Yes	214	60	1	
	No	24	10	0.67 (0.31, 1.49)	
ART	Pre-ART	143	45	1	
	On ART	95	25	1.19 (0.69, 2.08)	
WHO stage	III	154	34	1	
	IV	84	36	0.52 (0.30, 0.88)	
TB treatment history	Yes	59	18	0.95 (0.52, 1.76)	
	No	179	52	1	
TB type	Pulmonary	175	44	1	
	Extra-pulmonary	63	26	0.61 (0.35, 1.07)	
TB Treatment side effect	Yes	10	8	0.34 (0.13, 0.89)	0.35 (0.12, 0.98) *
	No	228	62	1	1

*significant at *p*-value <0.05

## Discussion

Tuberculosis and HIV co-infection is one of the most public health challenges in sub-Saharan Africa [18]. It is recognized that both TB and HIV contributes to each other’s progress [19] that affects the successful outcome of TB treatment, which ranges from 28.9% in Ethiopia [20] to 84.17% in India [21] and 86.0% in Malawi [22].

The overall successful TB treatment outcome in this study was 77.3%. This result is higher than studies conducted in India 67.2% [23], Iran 64.0% [24], Malaysia 53.4% [25], Ghana 64.0% [26], Nigeria 48.8% [27] and 62.7% [28], and Ethiopia 28.9% [20], 60.7% [29], 70.8% [17]. The possible explanation for this discrepancy might be associated with the difference in number of study participants involved in the study. On top of that, the handling of transfer out cases as one of the case definition of the TB treatment outcome in various studies indicated earlier may have also an effect on the reduction of the successful treatment outcome. But, in our cases, we did not include transferred out as one of the TB treatment outcome. Successful Tb treatment outcome may also be affected by the presence of more deaths and/or defaulters. It is evidenced by the studies conducted in Ethiopia where 11.4% transferred out, 10.0% defaulters and 17.4% deaths were recorded [29]; in Malaysia, 25.6% of the study participants were defaulters and the other 21.0% were death [25], which accounted a total of 46.6%.

The successful TB treatment outcome which we found in the current study is nearly in agreement with studies conducted in Asian countries like in Indian; 75.0% [30], 79.0% [31], and 80.0% [32]; in Vietnam 74.0% [33]; in Africa, Zambia 80.0% [34] and Ethiopia 80.5% [35]. However, it is lower than the target of successful TB treatment outcome (85%) recommended by the WHO [9]. Furthermore, the successful outcome of TB treatment found in India was 84.17% [21], Malawi 86% [22]; and in South Africa 82.2% [36] which are higher than the outcome we found in the current study. Of course, the current finding showed substantial improvement compared to the two studies conducted in Ethiopia that ranges from 28.9 to 70.8% [17, 20].

The variation between our finding and results of others might be due to the presence of TB and HIV drug-drug interaction, patient’s awareness on importance of adhering to TB treatment, and availability of facilities used to screen or diagnose TB in HIV/AIDS patients. In addition, a patient who is on TB-ART co-treatment will have higher pill burden and most likely will experience more side effects compared to those patients who are infected with TB only. These factors may lead to have low adherence to anti-TB drugs [37]. In addition, the substantial figures of defaulters and death recorded, and socio-economic conditions of the society in our cases may contribute for the low treatment outcome. These may support the reasons for many countries that are failing to achieve adequate successful TB treatment outcomes.

Being residing in the Gondar town is found to be significantly associated with the successful TB treatment outcome in our study which is in line with a study conducted in the same country [17, 38]. The possible explanation for this condition is that being residing in the town might be positively associated with TB notification [39, 40] which lead to the successful TB treatment outcome of the patients. Secondly, it is evidenced by a study conducted in Jimma, Ethiopia, where a rural resident had lower TB treatment outcome [41]. In addition, a better awareness and information about TB and HIV/AIDS might be more in the town than outside. Lastly, availability of health institutions at near to the vicinity in the town and fear of stigma and discrimination in outside of the town might be contributed for the successfulness as well as unsuccessfulness of TB treatment outcome.

Our finding revealed the presence of a significant association between mean baseline body weight < 43.7 kg measured at the commencement of TB treatment and the unsuccessful of TB treatment outcome. It is supported by a finding reported in Sothern Ethiopia in which survival of TB patients is lowered when one is HIV positive and has low baseline body weight during the initiation of TB treatment [1]. Another two studies conducted in Ethiopia also supported our finding, in which lower body weight of the patients at the initiation of TB treatment was significantly associated with unsuccessful outcome of TB treatment [42], and body weight at the initiation of TB treatment (<35 kg) was a significant risk factor for death at the time of TB treatment [43].

Like documented in the literature [35], finding of this study also showed that those co-infected patients who were on bedridden is significantly associated with the unsuccessful TB treatment outcome compared to those who were in playing and/or working (or active) condition. It is also consistent with a study conducted in Bair Dar, Ethiopia where more TB/HIV co-infected bedridden patients had unsuccessful TB treatment outcome or an increased risk of mortality during TB treatment [2]. In addition, as indicated in various literatures, being in bed ridden condition of the functional status among TB/HIV co-infected patients demonstrated unsuccessful TB treatment outcome [41, 44].

The possible explanation for this might be AIDS patients are likely to become malnourished from constantly being sick, from diarrhea that prevents absorption of nutrients, from loss of appetite and sores of mouth that make eating difficult, and from opportunistic infections. Similarly, weight loss and bedridden in TB patients might be explained partly by the loss of appetite and loss of energy by the disease itself and being immovable or inactive. Those patients who are in the very low body weight and bed ridden might be unable to work and care for themselves, live at home, and requires varying amount of assistance which may equivalent to institutional or hospital care from others and may lead to the progression of severity of diseases and even to death.

In this study, patients who had treatment side effect showed unsuccessful TB treatment outcome compared to those who had not experience the treatment side effect. This is in line with various studies in which they found that being co-infected with TB/HIV is significantly associated with unsuccessful treatment outcome [45, 46]. The possible reason could be related to pill burden, increase in adverse effect, drug-to-drug interaction, and drug induced toxicity, which reduces the adherence of the patients to their TB treatment regimen.

Apart from such important findings, this study is not without limitation. As common for secondary data based studies, important variables like CD4 level, distance to the health facility, and weight of the patients were incomplete from the records other than the baseline measures. In addition, this study may suffer from low sample size because of lack of complete patients’ medical record.

## Conclusion

This study showed that the successful anti-TB treatment outcome among TB/HIV co-infected patients who attended their treatment at the Gondar University Hospital TB DOTS clinic was below the target set by World Health Organization (WHO). Being residing in the Gondar town, having baseline body weight above the mean, able to perform usual work in or out of the house, and being free of treatment side effects were found to be factors associated with successful TB treatment outcome. Thus, we recommended that strengthening the integration of TB/HIV collaborative activities could improve the treatment outcome. In addition, improvise means of TB patients’ follow up and tracing to keep them in contact with health professionals at service delivery point during treatment could increase the successfulness of TB treatment outcome.

## Abbrevations

AFB: Acid fast bacilli
AIDS: Acquired immune deficiency syndrome
AOR: Adjusted odds ratio
ART: Antiretroviral therapy
CI: Confidence interval
COR: Crude odds ratio
CPT: Co-trimoxazole prophylactic treatment
DOTS: Directly observed treatment short course
HIV: Human immunodeficiency virus
INH: Isoniazid
PTB: Pulmonary tuberculosis
SPSS: Statistical package for social sciences
TB: Tuberculosis
WHO: World Health Organization
